# Missingness mechanisms and generalizability of patient reported outcome measures in colorectal cancer survivors – assessing the reasonableness of the “missing completely at random” assumption

**DOI:** 10.1186/s12874-024-02236-z

**Published:** 2024-05-03

**Authors:** Johanne Dam Lyhne, Allan ‘Ben’ Smith, Lars Henrik Jensen, Torben Frøstrup Hansen, Lisbeth Frostholm, Signe Timm

**Affiliations:** 1 Department of Oncology, University Hospital of Southern Denmark, Beriderbakken 4, Vejle, 7100 Denmark; 2 The Daffodil Centre, The University of Sydney, A Joint Venture with Cancer Council NSW, Sydney, NSW Australia; 3 Research Clinic for Functional Disorders and Psychosomatics, Aarhus University Hospital, Aarhus, Denmark; 4 Department of Clinical Medicine, Aarhus University, Aarhus, Denmark

**Keywords:** Patient reported Outcome, Colorectal cancer, Registries, Survivors, Missing data, Non-responders

## Abstract

**Background:**

Patient-Reported Outcome Measures (PROM) provide important information, however, missing PROM data threaten the interpretability and generalizability of findings by introducing potential bias. This study aims to provide insight into missingness mechanisms and inform future researchers on generalizability and possible methodological solutions to overcome missing PROM data problems during data collection and statistical analyses.

**Methods:**

We identified 10,236 colorectal cancer survivors (CRCs) above 18y, diagnosed between 2014 and 2018 through the Danish Clinical Registries. We invited a random 20% (2,097) to participate in a national survey in May 2023. We distributed reminder e-mails at day 10 and day 20, and compared *Initial Responders* (response day 0–9), *Subsequent Responders* (response day 10–28) and *Non-responders* (no response after 28 days) in demographic and cancer-related characteristics and PROM-scores using linear regression.

**Results:**

Of the 2,097 CRCs, 1,188 responded (57%). Of these, 142 (7%) were excluded leaving 1,955 eligible CRCs. 628 (32%) were categorized as *initial responders*, 418 (21%) as *subsequent responders*, and 909 (47%) as *non-responders.* Differences in demographic and cancer-related characteristics between the three groups were minor and PROM-scores only marginally differed between initial and subsequent responders.

**Conclusion:**

In this study of long-term colorectal cancer survivors, we showed that initial responders, subsequent responders, and non-responders exhibit comparable demographic and cancer-related characteristics. Among respondents, Patient-Reported Outcome Measures were also similar, indicating generalizability. Assuming Patient-Reported Outcome Measures of subsequent responders represent answers by the non-responders (would they be available), it may be reasonable to judge the missingness mechanism as Missing Completely At Random.

## Background

Due to advancements in medical science, increasing numbers of people are living with and after colorectal cancer worldwide [1]. The value of new treatments is no longer isolated to how long you survive, but also how well you survive [2, 3]. Quality of life measures are necessary to include as primary or at least secondary outcomes in interventional research [4–6].

Patient Reported Outcomes (PROs) [7, 8] have gained attention in healthcare as a way to gather information directly from patients about their health conditions. Patient-Reported Outcome Measures (PROMs) are specific tools used to assess PROs, often through self-completed questionnaires [8]. PROMs provide valuable insights into the patient’s perspective on treatment-related issues, functional abilities, and quality of life [9]. However, the nature of PROMs introduces certain challenges related to data collection [10], as they rely on patient willingness and ability to respond, hence the response can be affected by various factors such as illness severity and study design [11].

Missing data is a frequent problem in studies using PROMs [12, 13], and is often adressed by conducting complete-case analyses and ignoring the missing data [14]. However, this method may lead to biased results, reduced statistical power to detect differences between treatments, limited generalizability and misleading conclusions [13, 15–19].

In studies with missing data (> 5%) possible statistical solutions depend on the missingness mechanisms [16]. There are three missingness mechanisms: missing at random (MAR), missing not at random (MNAR) and missing completely at random (MCAR) [15, 16, 20]. If the missingness mechanism and hence the probability of response depend on the observed data then data is said to be MAR. In case of MAR, missing data may be addressed using i.e. multiple imputation to predict the missing values based on observed data. If the probability of response depends on both observed data and missing data then data is said to be MNAR, and may be adressed by conducting sensitivity analyses with best-and-worst case scenarios. If the probability of response is not associated with either the observed or missing values, data is said to be MCAR - and responders are considered representative of non-responders and complete-case analyses may not cause bias, but only enlarged standard errors due to the reduced statistical power.

In a study using PROM scores as an outcome then MCAR means the PROM value may not differ between responders and non-responders. Likewise, responders and non-responders may not differ with regard to other data observed. The MCAR assumption is hard to prove given that the PROMs are actually missing, however using a dataset collected with repeating reminders of the patients to respond may shed light upon the nature of missing PROMs. Comparing responders, who respond following the first invitation (hereafter denoted “Initial responders”) to the responders who were actually non-responders until 2nd or 3rd invitation but replyed subsequently (hereafter denoted “Subsequent responders”) creates an opportunity to evaluate the PROMs and characterize the patients who were non-responders at first, and to assess if the MCAR assumption may be reasonable. Comparing registry data between responders and non-responders provides insights into the generalizability of the respondents.

Acknowledging the issues with PROMs, including handling of missing data and related statistical approaches, we aimed to investigate the generalizability of respondents in a random sample of 20% of our registry-based national cohort of > 10,000 long-term colorectal cancer survivors (CRC) before continuing with a large cross-sectional study [21] on prevalence of symptoms indicative of psycho-oncological late effects and quality of life.

We assessed missingness mechanisms of PROM-data among initial responders and subsequent responders. We assumed, that PROM-data from subsequent responders may be indicative of PROM-data from non-responders, had the subsequent responder not been prompted with several reminders, and hence give insight into the missing data of the non-responders. Furthermore, we addressed the generalizability of all responders compared to non-responders by comparing clinical and demographic characteristics extracted from national registries. The study will provide insight into the missingness mechanisms in the setting of a nationwide cross-sectional study and add to the literature on issues with generalizability and possible methodological solutions to overcome missing data problems during statistical analyses. By addressing this issue associated with PROMs, researchers can better understand how to collect, interpret and utilize PROM data in future studies.

## Method

This study complies with STROBE guidelines [22] for reporting observational studies in epidemiology. The data presented in this study is drawn from a population-based cross sectional study of “Late Effects After Colorectal Cancer” conducted among Danish long-term (4–10 years post initial treatment) CRCs, which is also being used for recruitment to a randomized controlled trial (RCT) of an online psychological intervention, see published protocol [21].

### Study population

We identified eligible CRCs through the Danish Colorectal Cancer Group (DCCG) [23] database hosted by The Danish Clinical Registries (RKKP) with no need to enter confidential electronic patient records. We invited all Danish CRCs above age 18, able to read and understand Danish, who have completed curative-intent cancer treatment with surgery and/or radiation and/or adjuvant chemotherapy between March 2014 (when the national Danish Colorectal Cancer Screening Program was launched) and December 2018, to participate.

### Data collection and definition of responders

Invited participants were a random 20% sample of the total cohort. We invited participants to answer an electronic questionnaire to screen for late effects. We distributed surveys through REDCap using the Danish national-wide secure, personal electronic mail box (e-Boks) between May 8th 2023 and May 12th 2023. In case of no answer on Day 10, the survey was automatically re-distributed. In case of no answer of Day 20, the survey was again re-distributed. *Initial responders* were defined as responders completing the questionnaire in the first 10 days following the initial invitation. *Subsequent responders* were defined as responders completing the questionnaire following either first or second reminder (10–28 days after initial invitation). *Non-responders* were defined as responders not completing the questionnaire within 4 weeks after last reminder. No incentives were offered.

The e-Boks system offers the option to set up automatic SMS/text message notifications whenever an email is received. Since this feature is personalized, we do not have specific data on its usage. No other phone calls or SMS/text messages were utilized to remind participants to respond.

### Patient reported outcome measures (PROMs)

The selected PROMs aim to capture the diversity in psycho-oncological late effects experienced by colorectal cancer survivors. Participants completed an 83-item survey (see Table 1) comprising six questionnaires validated in Danish:

1. **The Fear of Cancer Recurrence Inventory– Short Form (FCRI-SF)** [24, 25] measures severity of fear of cancer recurrence on a 9-item subscale with response categories on a 5-point Likert like scale ranging from 0 to 4. Scale scores range from 0 to 36 with higher scores indicating greater severity.
2. **The Fear of Cancer Recurrence – 1 revised (FCR-1r)** [26] measures fear of cancer recurrence on a single item ranging from 0 to 10. Higher score indicates greater severity.
3. **Symptom Checklist-90-R** (SCL) [27] subscales [28] measuring anxiety (**SCL-anx**) in 4 items, depression (**SCL-dep**) in 6 items and emotional distress (**SCL-distress**) in 8 items on a 5-point Likert like scale rating from 0 to 4 resulting in simple sum scores ranging from 0 to 16, 24 and 32 respectively.
4. **The Whiteley-6** [29, 30] measures health anxiety on a 5-point Likert like scale with a simple sum score ranging from 0 to 24.
5. The **EQ-5D-5 L** and **EQ VAS** scale [31] measures health state in five dimensions: mobility, self-care, usual activities, pain/discomfort and anxiety/depression on 5 levels resulting in a 5-digit number that describes the patient’s health state. The VAS targets health state today and ranges from 0 to 100. EQ-5D-5 L was analysed as sumscore index value and standardized according to the US EQ-5D-5L value set as recommended by EuroQoL [32].
6. **The BDS Checklist** [33] measures physical symptom load on a 5-point Likert like scale with a simple sum score rating from 0 to 100. Higher scores indicates greater symptom load. The 25-item questionnaire covers symptoms related to cardio/pulmonary function, bowel function, musculoskeletal function and general function. The BDS Checklist’s conceptualization for diagnosing bodily distress syndrome (BDS) was not applied since this was a cross-sectional survey with no participant contact. A high score on physical parameters can then represent comorbidity, late effects or functional disorders.

The BDS Checklist was supplemented by nine items covering well known late effects after colorectal cancer not included in the checklist. Three items relate to bowel function (Involuntary passage of air and/or loose stools, difficulty emptying the bowels during toilet visits and urgent bowel movement), five items relate to urinary function (Involuntary bladder leakage, bleeding from the bladder, frequent urination, difficulty emptying the bladder and bladder pain) and one item addresses impaired sexual function. Response categories on all additional items were on a 5-point Likert like scale similar the BDS Checklist, and the scale score range for the individual organ systems is shown in Table 1.

Quality of life was rated using a VAS scale (QoL VAS) inspired by the EuroQoL Health VAS scale, as the word “health” was changed into “Quality of life”. Additionally, the survivors were asked to report demographic and cancer-related outcomes supplementing the data drawn from registries.

In case of missing single items within the above mentioned questionnaires, the item was replaced with a 0 indicating a conservative approach, assuming that the symptom in question was not present.

### Data cleaning

**Fig. 1:**
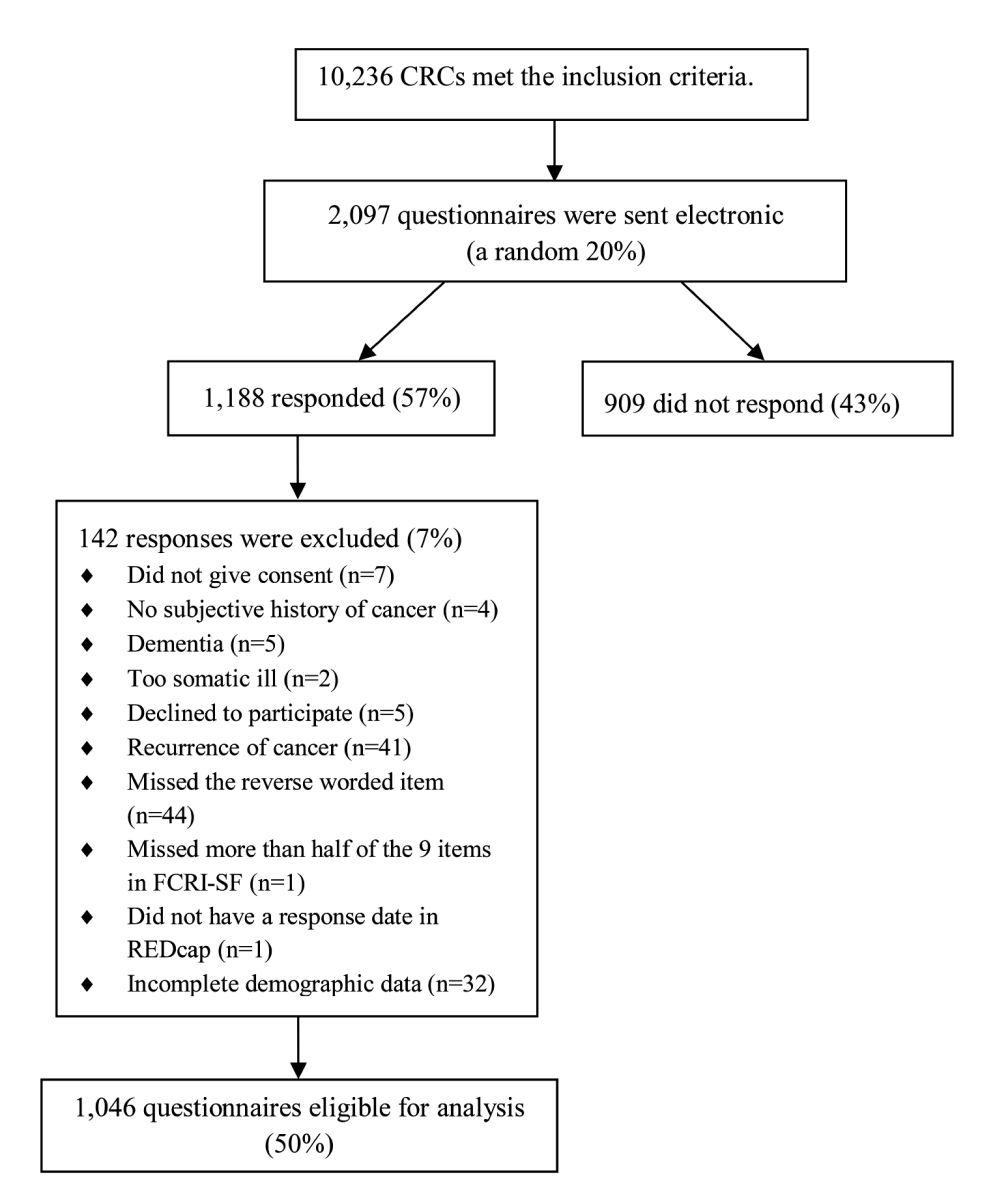
Participant flow chart

Returned questionnaires were excluded in case of cancer recurrence or if “dementia”, “terminal/too somatic ill” or “dead” were communicated by caregivers to the primary investigator by telephone or email. In certain instances, survivors indicated “no memory of cancer” or “do not want to participate”, leading to their exclusion. Similarly, responses lacking consent to participate were excluded. Cases that missed the reversed wording of item 5 on the FCRI-SF and presented with a response pattern of “0” were excluded, as this was interpreted as inattention or lack of motivation [34], see Fig. 1. Only cases with complete demographic data were analyzed, hence cases with missing items were also excluded.

### Statistics

We compared demographic and cancer-related group characteristics (i.e., initial vs. subsequent vs. non-responders) using balance diagnostics and reported as standardized differences accompanied by descriptive statistics. An absolute standardized difference of > 0.1 was interpreted as imbalance between groups [35]. Cancer-related characteristics were compared using Chi-squared test and reported as absolute numbers, proportions and p-values. PROM-scores were analyzed using generalized linear regression models and reported as β-coefficients, 95% CI and p-values for subsequent responders using initial responders as the reference. Assumptions of normality, linearity and homoscedasticity were checked and found to be reasonable.

Analyses were performed on responders with complete PROMs and all non-responders (in total *N* = 1,955). Statistical analyses were performed in Stata 17 (Stata Corp., College Station, TX, USA).

## Results

A total of 10,236 participants were diagnosed with colorectal cancer and treated with curative intend in the period March 2014 to December 2018. Of this cohort, 20% (2,097) were randomly selected for inclusion in this cross-sectional study and emailed a questionnaire between May 8th and May 12th 2023.

Of the 2,097 eligible CRCs, 1,188 responded (57%), see Fig. 1. Of these, 142 (7%) were excluded due to comorbidity (*n* = 7), unwillingness to participate/no history of cancer (*n* = 16), recurrence (*n* = 41) or low quality/incomplete data (*n* = 78) resulting in 1,955 eligible CRCs.

628 respondents (32%) were categorized as *initial responders* and 418 respondents (21%) as *subsequent responders.* There were 909 (47%) *non-responders*.

### Response pattern

**Fig. 2:**
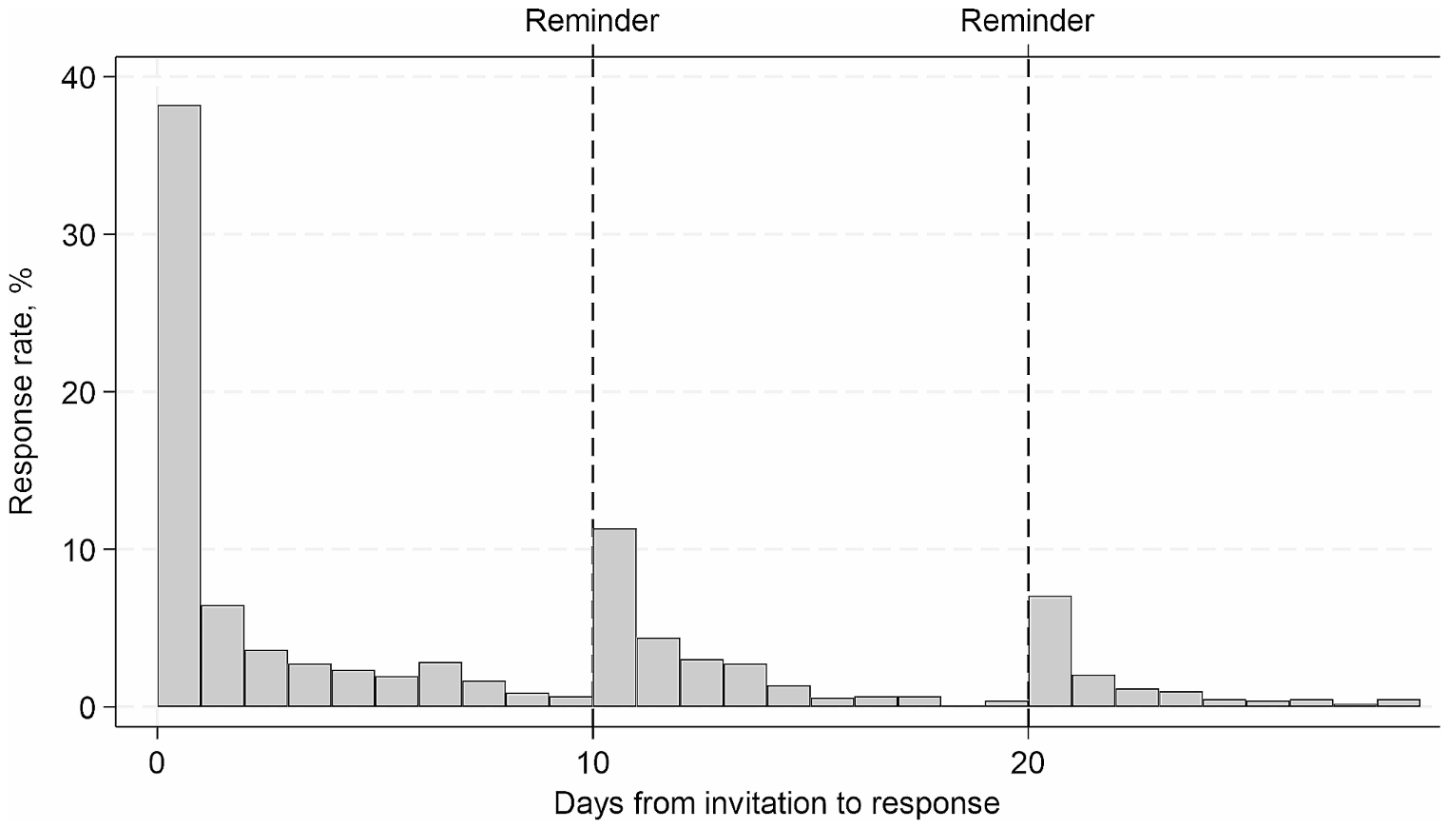
Responses divided on days. Day 0 equals the distribution day. Reminders are send on day 10 and day 20 (vertical lines)

37% responded on Day 0 and 6% on Day 1. Response rates Day 2–9 were < 4%. A similar pattern was seen after 1st reminder on Day 10 (11%) and 2nd reminder at Day 20 (7%) see Fig. 2.

### Differences in demographic characteristics

We observed minor differences in demographic characteristics. Non-responders tend to be marginally older than initial and subsequent responders (median age 75 vs. 73 and 71, standardized difference 0.18 and 0.16, respectively), and consequently a larger proportion of the subsequent responders were employed (27% vs. 23%, standardized difference 0.18), see Table 2. The initial and subsequent responders were similar in terms of education, sex, marital status, language, ethnicity and children.

### Differences in cancer-related characteristics

We observed no major differences in cancer-related characteristics between initial-responders, subsequent-responders and non-responders. A marginally higher proportion of both initial and subsequent responders, who were eligible for enrollment in the national colorectal cancer screening program (aged 50–75 at the time of diagnosis) had their cancer detected through screening as opposed to non-responders (45% and 43% compared to 39%). However, no disparity was observed in terms of T-category. N-category differed between groups mainly due to an uneven distribution of missing data, see Table 3.

### Differences in PROMs

Concerning the physical and psychological parameters, only the single-item PROMs (the FCR-1r and sexual function) differed between initial responders and subsequent responders, as subsequent responders reported slightly higher FCR-1r (β 0.45, 95% CI(0.13–0.78)) and lower sexual dysfunction (β -0.24, 95% CI(-0.41 – -0.08)). We observed no difference in quality of life, health state, symptoms of anxiety, health anxiety, depression, or physical symptoms in general, see Table 4. As psychological parameters were measured through PROMs, we do not have data on non-responders.

## Discussion

This study analysed generalizability and missingness mechanisms of PROMs in a random sample of long-term colorectal cancer survivors. Based on the present data we report a high grade of comparability between initial responders, subsequent responders and non-responders concerning demographic and cancer-related characteristics. In addition, initial and subsequent responders showed similar outcomes on PROMs, suggesting that the mechanisms of missing data among non-responders in this population can be assumed to be missing completely at random.

In research, high response rates are desirable to enhance validity and generalizability, and several strategies to enhance PRO response rates [10, 11, 13, 36–40] and deal with missing data have been developed [16, 20, 41]. However, potential bias does not depend on the response rate, but on the degree of similarity between respondents and non-respondents [42]. Achieving high response rates increases the likelihood of similarity, but often requires significant resources for reminders (either postal mail, email or phone call) and may also be burdensome for the participants.

If reminders are to be distributed, the data collecting process can be accelerated, as the response pattern of this population reveals a rapid decline in response rates similar to previous research [39], dropping below 1% within a week.

The findings in this study align with previous research demonstrating limited evidence of non-response bias. In a population-based survey of childhood, adolescent and young adult Norwegian long-term cancer survivors (the NOR-CAYACS study) [43] and in children participating in the Swiss Childhood Cancer survivor study [44] the authors found no evidence of relevant non-response bias in any of the investigated outcomes when comparing observed prevalence in respondents to expected prevalence in a constructed total population. Both studies were performed with postal surveys, and a long period between initial invitation and reminders (up to five months), which do not represent the electronic era of today.

In contrast to our findings, observational data from the Dutch PROFILES study, which included a heterogeneous cancer population, found respondents to be healthier than the population of interest, despite achieving a high response rate of 69% [45]. Similarly, in a longitudinal examination of an American breast cancer population using PROMs non-responders tended to be older and more frequently identified as non-English speaking, of Hispanic ethnicity, or of Black race compared to responders. Consequently, the authors concluded that the PROM results may not accurately represent the experiences of the entire American breast cancer population [46]. Downing et al. also found nonwhite ethnicity and those living in the most socioeconomically deprived areas less likely to participate in a population-level study, and concluded that the results presented may underestimate the true impact of colorectal cancer on health related quality of life [47]. The Danish population of CRCs is relatively homogeneous, and the current study was not designed to specifically investigate the influence of language, socioeconomic status or ethnicity.

While the findings of this study cannot be extrapolated to clinical cancer surveillance in general, understanding the characteristics of patients who do not respond to PROMs is essential especially given the current trend in survivorship care, which emphasizes a patient-centered approach, encouraging patients to self-report symptoms of recurrence, side effects, or late effects as they arise [48]. Based on this study, no explicit demographic or cancer-related characterization of this group can be made.

### Strengths and limitations

A significant strength of this study lies in the comprehensive dataset available from the DCCG database, which includes valuable background variables and clinical information for all participants. With a database completeness exceeding 95%, we were provided with a unique opportunity to compare responders and non-responders. By recruiting study participants from this registry, we ensured the assembly of a complete and unbiased sample, allowing for a thorough examination of generalizability.

Nonetheless, we acknowledge certain limitations. Given the cross-sectional study design, PROM-data from non-responders are unavailable, and hence PROM-responses from subsequent responders, who were non-responders until distribution of reminders, may be the closest available. The assumption that subsequent responders may be indicative of non-responders cannot be tested. Multiple PRO scores from different assessment time points would add information regarding the nature of missingness of subsequent time points. Nevertheless, this will still not add information on never-responders.

Whenever missing data is an issue, study dependent missingness mechanisms should be explored and handled accordingly taking the specific research question, outcomes, exposures, covariates etc. into account. Therefore, more general advice on specific imputation strategies cannot be made based on this study. However, this study may help to inform the decision on how to handle missing data in similar populations or study designs.

Another limitation pertains to the methods of data registration, as data on for example lifestyle factors, comorbidity, and performance are recorded by the primary surgeon within 30 days after surgery, potentially introducing recall bias. Another limitation of this study is that the three survey invitations do not have individualized links. Consequently, a person may have responded to the initial invitation on day 11 without a reminder. The generalizability to other cancer diagnoses cannot be determined based on our findings.

## Conclusion

In this study of long-term colorectal cancer survivors, we showed that initial responders, subsequent responders, and non-responders exhibit comparable demographic and cancer-related characteristics. Among respondents, PROMs were also similar, indicating generalizability.

Assuming PROMs of subsequent responders represents PROMs of the non-responders (would they be available), as suggested by our analysis, it may be reasonable to judge the missingness mechanism as Missing Completely At Random. Hence, imputation methods may be an option to enhance statistical power.

**Table 1:** Data sources

Data source	Data	Parameter	Validated PROM	Items
REGISTRY	Demographic	Age		1
		Sex		1
	Clinical	Cancer type		1
		Cancer stage		1
		Ostomy		1
		Screened to diagnosis		1
		WHO performance status		1
		Time since surgery		1
PRO	Demographic	Marital status		1
		Employment status		1
		Education		1
		Citizenship		1
		Language		1
		Children		1
	Clinical	Cancer recurrence_a_		1
		Chemotherapy_a_		1
		Radiation_a_		1
	Psychological	Health state	EQ-5D-5L_a_	5
			EQ-VAS_a_	1
		Quality of life	QoL-VAS_a_	1
		Fear of cancer recurrence	FCRI-SF_a_	9
			FCR-1r_a_	1
		Health Anxiety	Whiteley-6	6
		Anxiety	SCL-4	4
		Depression	SCL-6	6
		Emotional distress	SCL-8	8
	Physical	Cardiopulmonary	BDS Checklist	6
		Bowel	BDS Checklist	10
		Musculoskeletal	BDS Checklist	7
		Urinay		6
		Sexual		1
		General symptoms	BDS Checklist	5

_a_ : Mandatory items

**Table 2:** Demographic characteristics of the initial responders, subsequent responders and non-responders (*N* = 1,955) compared using balance diagnostics. Standardized differences > 0.1 indicate imbalance between groups

			Initial responders	Subsequent responders	Non-responders_a_	Standardized difference
Survivors, N (% of source population)			628 (32%)	418 (21%)	909 (47%)	
Age (median, min; max)			73, 43;91	71, 38;94	75, 29;97	0.18_b_, 0.16_c_
Sex, N (%female)			260 (41%)	191 (46%)	375 (41%)	0.09_b_, 0.01_c_
Years since diagnosis (median, min; max)			6.7, 4.4;9.2	6.8, 4.4;9.2	6.9, 4.4;9.2	0.04_b_, 0.05_c_
Marital status, N(%)						0.09
	Married		420 (67%)	288 (69%)	-	
	Not married		44 (7%)	33 (8%)	-	
	Divorced/seperated		45 (7%)	30 (7%)	-	
	Widowed		85 (14%)	44 (11%)	-	
	Living together		34 (5%)	23 (5%)	-	
Children, N(%yes)			560 (89%)	377 (90%)	-	0.03
Employment status, N(%)						0.18
	Employed		147 (23%)	113 (27%)	-	
	Has been employed		479 (76%)	297 (71%)	-	
	Has never been employed		2 (1%)	8 (2%)	-	
Education at any level, N(%yes)			518 (82%)	339 (81%)	-	0.04
Born and raised in Denmark, N(% Yes)			613 (98%)	401 (96%)	-	0.10
Primary language, N(% Danish)			624 (99%)	411 (98%)	-	0.09

_a_ : The survivors were asked not to respond in case of cancer recurrence, and hence this group also includes survivors who are intentionel non-responders

_b_ : comparing initial responder to subsequent responders

_c_ : comparing initial responders to non-responders

**Table 3:** Cancer-related characteristics of initial responders, subsequent-responders and non-responders. P-values corresponds to chi-squared test

			Initial responders (*N* = 628)	Subsequent responders (*N* = 418)	Non-responders (*N* = 909)	*p*
Cancer diagnosed following screening, N(%yes_a_)			236 (45%)	150 (43%)	268 (39%)	0.07
Localization of tumor, N (%)						
	Colon		413 (66%)	272 (65%)	629 (69%)	0.21
	Rectum		215 (34%)	146 (35%)	280 (31%)	
T-category at diagnosis, N (%)						
	T0		9 (2%)	5 (1%)	10 (1%)	0.45
	T1		58 (9%)	37 (9%)	104 (11%)	
	T2		119 (19%)	82 (19%)	136 (15%)	
	T3		163 (26%)	104 (25%)	223 (25%)	
	T4		34 (5%)	25 (6%)	49 (5%)	
	Tx/missing_b_		245 (39%)	166 (40%)	387 (43%)	
N-category at diagnosis, N (%)						
	N0		216 (34%)	138 (33%)	289 (32%)	0.04
	N1		115 (18%)	48 (12%)	114 (13%)	
	N2		53 (9%)	47 (11%)	83 (9%)	
	Nx/missing_b_		244 (39%)	185 (44%)	423 (46%)	
Metastatic disease at diagnosis, N (%)						
	cM0		594 (95%)	394 (94%)	853 (94%)	0.92
	cM1		28 (4%)	19 (5%)	48 (5%)	
	cMx/missing		6 (1%)	5 (1%)	8 (1%)	
Performance status at surgery, N (%)						
	0–1		596 (95%)	391 (94%)	841 (93%)	0.11
	> 1		12 (2%)	9 (2%)	36 (4%)	
	Unknown		20 (3%)	18 (4%)	32 (3%)	
Chemotherapy, N(%yes)			223 (36%)	168 (40%)	-	0.13
Radiotherapy, N(%yes)			40 (6%)	35 (8%)	-	0.22
Stoma, N (%)						
	Yes (permanent)		2 (1%)	2 (1%)	8 (1%)	0.07
	Yes (temporary)		94 (15%)	66 (16%)	103 (11%)	
	No		532 (84%)	350 (83%)	798 (88%)	

_a_: “Yes” is annotated for individuals who submit a stool sample within three months after receiving screening invitation

_b_: data on TN-stage was not available before January 2016

**Table 4:** Generalized linear regression models comparing PROM-scores in initial responders (reference group) to subsequent responders (*N* = 1,046)

			Scale range(‘indicates best status)	Mean score for initial responders (REF.), const. (95% CI)	Mean difference between initial and subsequent responders, (95% CI)	*p*
Health state						
	EQ-5D-5 L		0–1’	0.87 (0.85 ; 0.88)	0.01 (-0.01 ; 0.03)	0.27
	EQ-5D VAS		0-100’	76.14 (74.70 ; 77.61)	1.21 (-1.10 ; 3.51)	0.31
Quality of life						
	QoL VAS		0-100’	78.68 (77.22 ; 80.14)	0.46 (-1.84 ; 2.77)	0.69
Fear of Cancer recurrence						
	FCR 1r		0’-10	2.57 (2.37 ; 2.78)	0.45 (0.13 ; 0.78)	0.01
	FCRI-SF		0’-36	10.07 (9.56 ; 10.58)	0.65 (-0.16 ; 1.46)	0.12
Anxiety and depression						
	SCL-4 (anxiety)		0’-16	1.08 (0.93 ; 1.23)	0.13 (-0.11 ; 0.36)	0.29
	SCL-6 (depression)		0’-24	1.30 (1.09 ; 1.51)	0.18 (-0.15 ; 0.51)	0.28
	SCL-8 (general distress)		0’-32	2.44 (2.13 ; 2.76)	0.30 (-0.20 ; 0.79)	0.24
	Whiteley-6 (health anxiety)		0’-24	2.69 (2.39 ; 2.98)	-0.07 (-0.53 ; 0.39)	0.77
Frequent physical symptoms						
	Cardio/pulmonary		0’-24	2.43 (2.18 ; 2.69)	0.14 (-0.27 ; 0.54)	0.51
	Bowel		0’-40	5.99 (5.52 ; 6.47)	0.28 (-0.47 ; 1.02)	0.47
	Musculosceltal		0’-28	4.16 (3.80 ; 4.53)	-0.01 (-0.60 ; 0.57)	0.97
	Urinary		0’-20	1.84 (1.66 ; 2.01)	-0.22 (-0.51 ; 0.06)	0.13
	Sexual		0’-4	1.03 (0.92 ; 1.14)	-0.24 (-0.41 ; -0.08)	0.01
	General		0’-20	3.20 (2.95 ; 3.45)	-0.09 (-0.49 ; 0.31)	0.66

## Data Availability

The data that support the findings of this study are available from the corresponding author upon reasonable request. Study data were collected and managed using REDCap electronic data capture tools hosted at Open Patient data Explorative Network (OPEN), Region of Southern Denmark.

## Abbreviations

CRC: Colorectal Cancer
FCR: Fear of Cancer recurrence
MAR: missing at random
MCAR: missing completely at random
MNAR: missing not at random
PROM: Patient Reported Outcome Measure

## References

[1] The global (2019). Regional, and national burden of colorectal cancer and its attributable risk factors in 195 countries and territories, 1990–2017: a systematic analysis for the global burden of Disease Study 2017, the lancet. Gastroenterol Hepatol.

[2] Kluetz PG, Slagle A, Papadopoulos EJ, Johnson LL, Donoghue M, Kwitkowski VE, Chen WH, Sridhara R, Farrell AT, Keegan P, Kim G, Pazdur R. Focusing on Core patient-reported outcomes in Cancer clinical trials: symptomatic adverse events, physical function, and Disease-related symptoms, clinical cancer research: an official journal of the American Association for Cancer Research, 22 (2016) 1553–8. 10.1158/1078-0432.CCR-15-2035 26758559

[3] Maspero M, Hull T (2023). Patient-reported outcomes in colorectal surgery. Clin Colon Rectal Surg.

[4] Mercieca-Bebber R, King MT, Calvert MJ, Stockler MR, Friedlander M (2018). The importance of patient-reported outcomes in clinical trials and strategies for future optimization. Patient Relat Outcome Measures.

[5] Giesinger JM, Efficace F, Aaronson N, Calvert M, Kyte D, Cottone F, Cella D, Gamper EM (2021). Past and current practice of patient-reported outcome measurement in Randomized Cancer clinical trials: a systematic review. Value Health: J Int Soc Pharmacoeconomics Outcomes Res.

[6] Au H-J, Ringash J, Brundage M, Palmer M, Richardson H, Meyer RM (2010). Added value of health-related quality of life measurement in cancer clinical trials: the experience of the NCIC CTG. Expert Rev PharmacoEcon Outcomes Res.

[7] Porter ME, Larsson S, Lee TH (2016). Standardizing patient outcomes measurement. N Engl J Med.

[8] Higgins TJ, Chandler JPT, Cumpston J, Li M, Page T, Welch MJ. VA, Cochrane Handbook for Systematic Reviews of Interventions version 6.3updated February (2022). Chapter 18: Patient-reported outcomes, Cochrane 2022, www.training.cochrane.org/handbook., (2022).

[9] I.S.f.Q.o.L, Research. Dictionary of quality of life and health outcomes measurement, Isoqol, Nancy Mayo, Milwaukee (WI), USA, (2015).

[10] Mercieca-Bebber R, Palmer MJ, Brundage M, Calvert M, Stockler MR, King MT (2016). Design, implementation and reporting strategies to reduce the instance and impact of missing patient-reported outcome (PRO) data: a systematic review. BMJ open.

[11] Palmer MJ, Mercieca-Bebber R, King M, Calvert M, Richardson H, Brundage M (2018). A systematic review and development of a classification framework for factors associated with missing patient-reported outcome data. Clin Trials.

[12] Galea S, Tracy M (2007). Participation rates in epidemiologic studies. Ann Epidemiol.

[13] Little RJ, D’Agostino R, Cohen ML, Dickersin K, Emerson SS, Farrar JT, Frangakis C, Hogan JW, Molenberghs G, Murphy SA, Neaton JD, Rotnitzky A, Scharfstein D, Shih WJ, Siegel JP, Stern H (2012). The prevention and treatment of missing data in clinical trials. N Engl J Med.

[14] Bell ML, Fiero M, Horton NJ, Hsu CH (2014). Handling missing data in RCTs; a review of the top medical journals. BMC Med Res Methodol.

[15] Sterne JA, White IR, Carlin JB, Spratt M, Royston P, Kenward MG, Wood AM, Carpenter JR (2009). Multiple imputation for missing data in epidemiological and clinical research: potential and pitfalls. BMJ.

[16] Jakobsen JC, Gluud C, Wetterslev J, Winkel P (2017). When and how should multiple imputation be used for handling missing data in randomised clinical trials - a practical guide with flowcharts. BMC Med Res Methodol.

[17] Fairclough DL, Peterson HF, Cella D, Bonomi P (1998). Comparison of several model-based methods for analysing incomplete quality of life data in cancer clinical trials. Stat Med.

[18] Fairclough DL, Peterson HF, Chang V (1998). Why are missing quality of life data a problem in clinical trials of cancer therapy?. Stat Med.

[19] Rosett HA, Locke SC, Wolf SP, Herring KW, Samsa GP, Troy JD, LeBlanc TW (2020). An analysis of missing items in real-world electronic patient reported outcomes data: implications for clinical care. Supportive care cancer: Official J Multinational Association Supportive Care Cancer.

[20] Dziura JD, Post LA, Zhao Q, Fu Z, Peduzzi P (2013). Strategies for dealing with missing data in clinical trials: from design to analysis. Yale J Biol Med.

[21] Lyhne JD, Smith AB, Frostholm L, Fink P, Jensen LH (2020). Study protocol: a randomized controlled trial comparing the efficacy of therapist guided internet-delivered cognitive therapy (TG-iConquerFear) with augmented treatment as usual in reducing fear of cancer recurrence in Danish colorectal cancer survivors. BMC Cancer.

[22] von Elm E, Altman DG, Egger M, Pocock SJ, Gøtzsche PC, Vandenbroucke JP (2007). The strengthening the reporting of Observational studies in Epidemiology (STROBE) statement: guidelines for reporting observational studies. Epidemiol (Cambridge Mass).

[23] Ingeholm P, Gögenur I, Iversen LH (2016). Dan Colorectal Cancer Group Database Clin Epidemiol.

[24] Simard S, Savard J (2009). Fear of Cancer Recurrence Inventory: development and initial validation of a multidimensional measure of fear of cancer recurrence. Supportive care cancer: Official J Multinational Association Supportive Care Cancer.

[25] Hovdenak Jakobsen I, Jeppesen MM, Simard S, Thaysen HV, Laurberg S, Juul T (2018). Initial validation of the Danish version of the fear of Cancer Recurrence Inventory (FCRI) in colorectal cancer patients. J cancer Survivorship: Res Pract.

[26] Smith A, Gao M, Tran M, Ftanou M, Jegathees S, Wu V, Jefford M, Lynch F, Dhillon HM, Shaw J, McDowell L, White A, Halloran C, Wiesenfeld D (2023). Bamgboje-Ayodele, evaluation of the validity and screening performance of a revised single-item fear of cancer recurrence screening measure (FCR-1r). Psycho-oncology.

[27] Olsen LR, Mortensen EL, Bech P (2004). The SCL-90 and SCL-90R versions validated by item response models in a Danish community sample. Acta Psychiatrica Scandinavica.

[28] Christensen K.S., Fink P., Toft T., Frostholm L., Ornbøl E., Olesen F. A brief case-finding questionnaire for common mental disorders: the CMDQ. Fam Pract. 2005;22:448–57. 10.1093/fampra/cmi025 15814580

[29] Veddegjærde KE, Sivertsen B, Wilhelmsen I, Skogen JC. Confirmatory factor analysis and item response theory analysis of the Whiteley Index. Results from a large population based study in Norway. The Hordaland Health Study (HUSK), Journal of psychosomatic research, 77 (2014) 213–218. 10.1016/j.jpsychores.2014.06.011 25149031

[30] Carstensen TBW, Ørnbøl E, Fink P, Pedersen MM, Jørgensen T, Dantoft TM, Benros ME, Frostholm L. Detection of illness worry in the general population: a specific item on illness rumination improves the Whiteley Index. J Psychosom Res. 2020;138:110245. 10.1016/j.jpsychores.2020.110245 32950761

[31] Rabin R, de Charro F. EQ-5D: a measure of health status from the EuroQol Group. Ann Med. 2001;33:337–43. 10.3109/07853890109002087 11491192

[32] Pickard AS, Law EH, Jiang R, Pullenayegum E, Shaw JW, Xie F, Oppe M, Boye KS, Chapman RH, Gong CL, Balch A, Busschbach JJV. United States Valuation of EQ-5D-5L Health States using an International Protocol, Value in health: the journal of the International Society for Pharmacoeconomics and Outcomes Research, 22 (2019) 931–41. 10.1016/j.jval.2019.02.009 31426935

[33] Budtz-Lilly A, Fink P, Ørnbøl E, Vestergaard M, Moth G, Christensen KS, Rosendal M (2015). A new questionnaire to identify bodily distress in primary care: the ‘BDS checklist’. J Psychosom Res.

[34] van Sonderen E, Sanderman R, Coyne JC (2013). Ineffectiveness of reverse wording of questionnaire items: let’s learn from cows in the rain. PLoS ONE.

[35] Austin PC (2009). Balance diagnostics for comparing the distribution of baseline covariates between treatment groups in propensity-score matched samples. Stat Med.

[36] Chowdhry AK, Gondi V, Pugh SL (2021). Missing Data in Clinical studies. Int J Radiat Oncol Biol Phys.

[37] Aiyegbusi OL, Roydhouse J, Rivera SC, Kamudoni P, Schache P, Wilson R, Stephens R, Calvert M (2022). Key considerations to reduce or address respondent burden in patient-reported outcome (PRO) data collection. Nat Commun.

[38] Papa N, Bensley JG, Hall K, Evans M, Millar JL (2022). Quantifying the effect email reminders have on patient reported outcome measure returns in a large prostate cancer registry. J Patient Rep Outcomes.

[39] Nielsen LK, King M, Möller S, Jarden M, Andersen CL, Frederiksen H, Gregersen H, Klostergaard A, Steffensen MS, Pedersen PT, Hinge M, Frederiksen M, Jensen BA, Helleberg C, Mylin AK, Abildgaard N (2020). Strategies to improve patient-reported outcome completion rates in longitudinal studies. Qual life Research: Int J Qual life Aspects Treat care Rehabilitation.

[40] Bulkley JE, O’Keeffe-Rosetti M, Wendel CS, Davis JV, Danforth KN, Harrison TN, Kwan ML, Munneke J, Brooks N, Grant M, Leo MC, Banegas M, Weinmann S, McMullen CK (2020). The effect of multiple recruitment contacts on response rates and patterns of missing data in a survey of bladder cancer survivors 6 months after cystectomy, quality of life research: an international journal of quality of life aspects of treatment. care Rehabilitation.

[41] Hamel JF, Sebille V, Le Neel T, Kubis G, Boyer FC, Hardouin JB (2017). What are the appropriate methods for analyzing patient-reported outcomes in randomized trials when data are missing?. Stat Methods Med Res.

[42] Groves RM (2006). Nonresponse rates and nonresponse bias in household surveys. Int J Public Opin Q.

[43] Lie HC, Rueegg CS, Fosså SD, Loge JH, Ruud E, Kiserud CE (2019). Limited evidence of non-response bias despite modest response rate in a nationwide survey of long-term cancer survivors-results from the NOR-CAYACS study. J cancer Survivorship: Res Pract.

[44] Rueegg CS, Gianinazzi ME, Michel G, Zwahlen M, von der Weid NX, Kuehni CE (2017). No evidence of response bias in a population-based childhood cancer survivor questionnaire survey - results from the Swiss Childhood Cancer Survivor Study. PLoS ONE.

[45] de Rooij BH, Ezendam NPM, Mols F, Vissers PAJ, Thong MSY, Vlooswijk CCP, Oerlemans S, Husson O, Horevoorts NJE (2018). Poll-Franse, Cancer survivors not participating in observational patient-reported outcome studies have a lower survival compared to participants: the population-based PROFILES registry. Qual life Research: Int J Qual life Aspects Treat care Rehabilitation.

[46] Srour MK, Tadros AB, Sevilimedu V, Nelson JA, Cracchiolo JR, McCready TM, Silva N, Moo TA, Morrow M (2022). Who are we missing: does Engagement in patient-reported outcome measures for breast Cancer Vary by Age, Race, or Disease Stage?. Ann Surg Oncol.

[47] Downing A, Morris EJ, Richards M, Corner J, Wright P, Sebag-Montefiore D, Finan P, Kind P, Wood C, Lawton S, Feltbower R, Wagland R, Vernon S, Thomas J, Glaser AW (2015). Health-related quality of life after colorectal cancer in England: a patient-reported outcomes study of individuals 12 to 36 months after diagnosis. J Clin Oncology: Official J Am Soc Clin Oncol.

[48] Hovdenak I, Thaysen HV, Bernstein IT, Christensen P, Hauberg A, Iversen LH, Johansen C, Larsen SL, Laurberg S, Madsen AH, Madsen MR, Rasmussen HV, Thorlacius-Ussing O, Juul T. Quality of life and symptom burden after rectal cancer surgery: a randomised controlled trial comparing patient-led versus standard follow-up. J cancer Survivorship: Res Pract, (2023). 10.1007/s11764-023-01410-4 PMC11424718 37395934

